# Validation of automated whole-body analysis of metabolic and morphological parameters from an integrated FDG-PET/MRI acquisition

**DOI:** 10.1038/s41598-020-62353-9

**Published:** 2020-03-24

**Authors:** P. Guglielmo, S. Ekström, R. Strand, R. Visvanathar, F. Malmberg, E. Johansson, M. J. Pereira, S. Skrtic, B. C. L. Carlsson, J. W. Eriksson, H. Ahlström, J. Kullberg

**Affiliations:** 1 Section of Radiology, Department of Surgical Sciences, Uppsala University, Uppsala, Sweden; 2 University of Milan Bicocca, Milan, Italy; 3 GE Healthcare, Chicago, USA; 4 Pharmaceutical Technology & Development, AstraZeneca AB, Gothenburg, Sweden; 5 Department of Medicine, Sahlgrenska University Hospital, Gothenburg, Sweden; 6 Early Clinical Development, Cardiovascular, Renal & Metabolism, IMED Biotech Unit, AstraZeneca, Gothenburg, Sweden; 7 Department of Medical Sciences, Clinical Diabetes and Metabolism, Uppsala University, Uppsala, Sweden; 8 Antaros Medical, Mölndal, Sweden

**Keywords:** Molecular medicine, Diabetes

## Abstract

Automated quantification of tissue morphology and tracer uptake in PET/MR images could streamline the analysis compared to traditional manual methods. To validate a single atlas image segmentation approach for automated assessment of tissue volume, fat content (FF) and glucose uptake (GU) from whole-body [^18^F]FDG-PET/MR images. Twelve subjects underwent whole-body [^18^F]FDG-PET/MRI during hyperinsulinemic-euglycemic clamp. Automated analysis of tissue volumes, FF and GU were achieved using image registration to a single atlas image with reference segmentations of 18 volume of interests (VOIs). Manual segmentations by an experienced radiologist were used as reference. Quantification accuracy was assessed with Dice scores, group comparisons and correlations. VOI Dice scores ranged from 0.93 to 0.32. Muscles, brain, VAT and liver showed the highest scores. Pancreas, large and small intestines demonstrated lower segmentation accuracy and poor correlations. Estimated tissue volumes differed significantly in 8 cases. Tissue FFs were often slightly but significantly overestimated. Satisfactory agreements were observed in most tissue GUs. Automated tissue identification and characterization using a single atlas segmentation performs well compared to manual segmentation in most tissues and will be valuable in future studies. In certain tissues, alternative quantification methods or improvements to the current approach is needed.

## Introduction

The increasing global prevalence of diabetes represent a significant public health problem^1^; a deeper understanding of underlying pathophysiological changes is necessary to enable a more preventive approach towards diabetes-related morbidity and mortality^2^. Recently, a new adult-onset diabetes classification has been proposed, comprising 5 clusters, which has proved to be superior in terms of prediction of disease progression, especially the development of diabetic complications, compared to the conventional categorization that provides a separation between type 1 (T1D) and type 2 diabetes (T2D)^3^. Reduced insulin sensitivity (IS) or “insulin resistance” (IR) is a hallmark feature of T2D and is associated with a significant increase in incidence and prevalence of cardiovascular disease (CVD) in patients with T2D^4^.

Positron emission tomography (PET) with [18F]-fluoro-deoxy-glucose (^18^F-FDG), a radiolabeled analogue of glucose, allows for noninvasive quantification of tissue specific glucose uptake during hyperinsulinemic euglycemic clamp (HEC), the gold standard technique which through the infusion of insulin and glucose determines the rate of body glucose disposal at a predetermined steady-state plasma glucose level^5^. It has previously been used in volume of interest (VOI)-based analyses in studies of assessment of the insulin-mediated tissue metabolism and whole-body IS (referred to as the M-value^6–11^). Magnetic resonance imaging (MRI) is a widely used tomographic imaging technique that without the use of ionizing radiation can support a wide range of medical diagnosis and research applications. MRI has also been extensively used to study body composition, in particular absolute quantification of fat mass and its relation to T2D^12^. In recent years with the advent of integrated PET/MR systems, new imaging capabilities are available for comprehensive metabolic studies and enable simultaneous measurement of IS, localization of glucose uptake and tissue composition, providing a comprehensive single assessment of the metabolic status of the subject^13^. Moreover, considering the lower radiation burden compared to a PET/computer tomography (CT) examination, it is acceptable to perform several whole-body investigations in the same subjects to observe longitudinal changes.

Imiomics is a new image analysis concept for automated analysis of whole-body MRI or PET/MR images^14^; this method uses an automated image registration process for voxel-wise normalization of whole-body image data to a reference volume (subject), i.e. displacement of the voxels to fit the corresponding voxels in the reference subject. Thus, creating a common coordinate system, enabling visualization and statistical analysis throughout the whole body of the entire cohort studied. The image registration produces a map in reference space, where each voxel contains the information of the deformation applied and the original signal intensity. This enables studies of differences in tissue volume, tissue fat content (FF) and the net uptake rate of [^18^F]FDG in tissues (Ki)^15^ between subjects. So far, Imiomics has demonstrated to be a promising tool in large-scale association studies, and in generation of a PET/MRI atlas of whole-body [^18^F]FDG scans from healthy subjects (unpublished data).

Manual image segmentation is one of the most tedious tasks in medical image processing. By utilizing Imiomics-registered images a single manual reference segmentation can be transformed to all other whole-body images in the cohort. This enables time-efficient analysis of all study subjects. Possible applications of this automated analysis concept include whole-body analysis of body composition including tissue volumetry and ectopic fat, assessment of glucose metabolism and efficient biodistribution analysis of new PET tracers. All of these tasks are very time-consuming when traditional approaches are applied and therefore not feasible in large-scale studies. Large databanks such as the UK Biobank (n = 100k scans)^16^ and the German national cohort (n = 30k scans)^17^ are great examples of where an automated image analysis concept such as Imiomics can be advantageously applied.

The purpose of this study was to validate the performance of Imiomics in combination with a single manual segmentation for automated assessment of tissue volume, fat content and glucose uptake from PET/MR images using manually performed segmentations as reference.

## Materials & Methods

### Subjects

This study included 12 randomly selected subjects from an ongoing type-2 diabetes study, where at the time, there were in total 35 subjects included. The subjects were recruited in three groups matched for age, gender and BMI. The groups consisted of control subjects, subjects with prediabetes and subjects diagnosed with T2D. Four subjects (2 males and 2 females) were randomly selected from each group.

Main study inclusion criteria were age (40–70 years) and BMI (25–35 kg/m2). Moreover, the T2D patients had to meet the following criteria: HbA1c 48–80 mmol/mol and no change in antidiabetic therapies during the last month. Basic characteristics of the included subjects are summarized in Table 1. The study was approved by the regional ethical committee, Uppsala, (application number 214/313), Sweden, and written informed consent was obtained from all subjects.

**Table 1:** Basic subject characteristics of the 12 randomly selected subjects.

	All		Controls		Prediabetics		T2D	
**Males/Females**	6/6		2/2		2/2		2/2	
**Age [years]**	62.8	(49–71)	60.5	(49–71)	64.0	(56–69)	63.8	(59–68)
**BMI [Kg/m**^**2**^**]**	30.9	(24.1–38.8)	29.3	(24.1–38.8)	33.2	(28.7–37.1)	30.4	(25–34.1)
**WC [cm]**	104.8	(82–123)	98.5	(82–112)	112.3	(102–123)	103.8	(90–116)
**FPG [mmol/L]**	5.7	(4.4–10.3)	5.0	(4.4–5.7)	6.1	(5.3–6.7)	8.6	(7.2–10.3)
**HbA1c [mmol/mol]**	42.8	(34–61)	34.0	(31–36)	37.8	(34–40)	56.5	(48–61)

BMI = Body Mass index, WC = Waist Circumference, FPG = Fasting Plasma Glucose. Data presented as mean (range).

### Hyperinsulinemic-euglycemic clamp

The hyperinsulinemic-euglycemic clamp was performed according to a previously well-established protocol described in more detail elsewhere^5,13^. All individuals were fasting overnight prior to the clamp-PET/MRI examination which was performed in the morning; furthermore, the subjects were instructed to avoid alcohol and caffeine intake for at least 6 hours, and intense physical activity for at least 24 hours before the examination.

The clamp, as achieved by a fix infusion of insulin and a variable infusion of glucose, allows for the assessment of an individual’s whole-body insulin sensitivity (M-value). This is achieved by measurement of the total glucose utilization of all tissues in the body. The M-value was calculated by dividing the glucose infusion rate during steady state (60–120 minutes after the initialization of the clamp) by lean body mass (mg/lbm kg/min) determined by Bioelectrical Impedance Analysis (BC-418, Tanita, Arlington Heights, IL). Semi-synthetic human insulin (Actrapid, Novo Nordisk, Copenhagen, Denmark) was infused at 56 mU/m^2^ body surface/min and a variable glucose infusion (200 mg/ml) was adjusted to maintain a stable plasma glucose level of 5.6 mmol/l. The data collection has previously been described in more detail^18,19^.

### PET/MR imaging

All subjects underwent simultaneous PET/MRI examinations on an integrated system (Signa PET/MR, GE Healthcare, Waukesha, WI). Dynamic PET data was collected to allow for voxel-wise quantification of [^18^F]FDG influx rate (Ki). One of the benefits over the commonly used standardized uptake value (SUV) analysis is the quantified Ki values in a tissue is less dependent of FDG uptake in other tissues. The imaging protocol has previously been described in detail^13^. In brief, an intravenous (i.v.) injection of 4 MBq [^18^F]FDG/kg bodyweight was used. A 10 min dynamic PET scan of the thorax was collected to capture early tracer dynamics of [^18^F]FDG^13,18^. After this, five whole-body PET scans (from head to toe) were acquired. The built-in solution for attenuation correction was used. This consisted of a 3D dual echo water fat separated MR scan (MRAC, TR = 4.0, Flip=5) that was collected at the start of each PET bed acquisition. The resolution of these water fat images was: 1.95 × 1.95 × 2.6 mm in sagittal × coronal × axial directions, respectively. Both the PET scans and the MRAC images were acquired in free breathing. All the necessary corrections were made for quantitative PET. The voxel-wise [^18^F]FDG influx rate (Ki) was calculated for the whole body using the Patlak method^15^. An image derived input function (IDIF) that was determined using a VOI in the aorta was used. The IDIF was corrected for blood cell bound radioactivity. PET data were analyzed using Matlab (Matlab 2015b, The Mathworks Inc, Natick, MA)^13^.

Dedicated scanning of liver and pancreas were also performed for state-of-the-art determination of tissue fat contents. This was performed by use of product 6-echo sequences^19^ integrated in the PET/MR system (IDEAL Quant). These also allow inclusion and thereby correction for signal decay (T2*) in the model. The scanning was performed using breath holds in exhaled position using axial slices without any angulation. Scan parameters for the liver and pancreas fat scan were: TR = 6.3, Flip=3, resolution 1.56 × 1.56 × 7.5 mm.

### Automated image analysis

Imiomics, as described by Strand *et al*.^14^, normalizes all subject data to a common reference coordinate system by using image registration. In brief, the registration process starts by registering bones, thereafter non-adipose tissue and last adipose-tissue, gradually allowing more elasticity in the deformation, based on the assumption that the skeleton is the tissue volume that varies the least between the subjects and fat volume the most. Here, a modified version of the Imiomics pipeline was used, replacing the registration method in the water-fat registration steps with a dense deformable registration method^20^. This allows for a transformation of higher resolution with point-to-point correspondences for all voxels as opposed to the previous method which relied on a low-resolution grid. The water and fat images from the MRAC of the last PET whole-body scan were used for the Imiomics analysis. These images allow calculation of absolute fat fraction images in which proton density fat fraction (pdff) concentrations, here denoted fat content (FF), can be measured in percentages in each voxel. The PET images were normalized using the same deformation as was produced for the water and fat images since MR and PET images were acquired simultaneously and therefore inherently co-registered.

A reference subject was selected based on BMI and visual quality control (male; 64 years old; BMI = 26.8 Kg/m^2^; control group) representing the common reference space. All remaining subjects were registered to the reference space using the pipeline. After the subject data had been registered to the common reference space, a single tissue VOI delineated in the reference space was used to extract the measurements for all registered subjects. Automated segmentations might contain both false positive and false negative voxels. False positive voxels were reduced by use of prior knowledge on expected tissue fat content. For brain, heart, liver, kidneys, intestines and muscles a criterion of <50% fat fraction was used filter the segmentation and thereby improving segmentation accuracy. For adipose tissue segmentations the criterion >50% fat fraction was used. Computing the Jacobian determinant of the deformation provides the local change in tissue volume caused by the registration. Using this information, the tissue volume in the original space could be quantified.

### Manual reference segmentation

**Figure 1:**
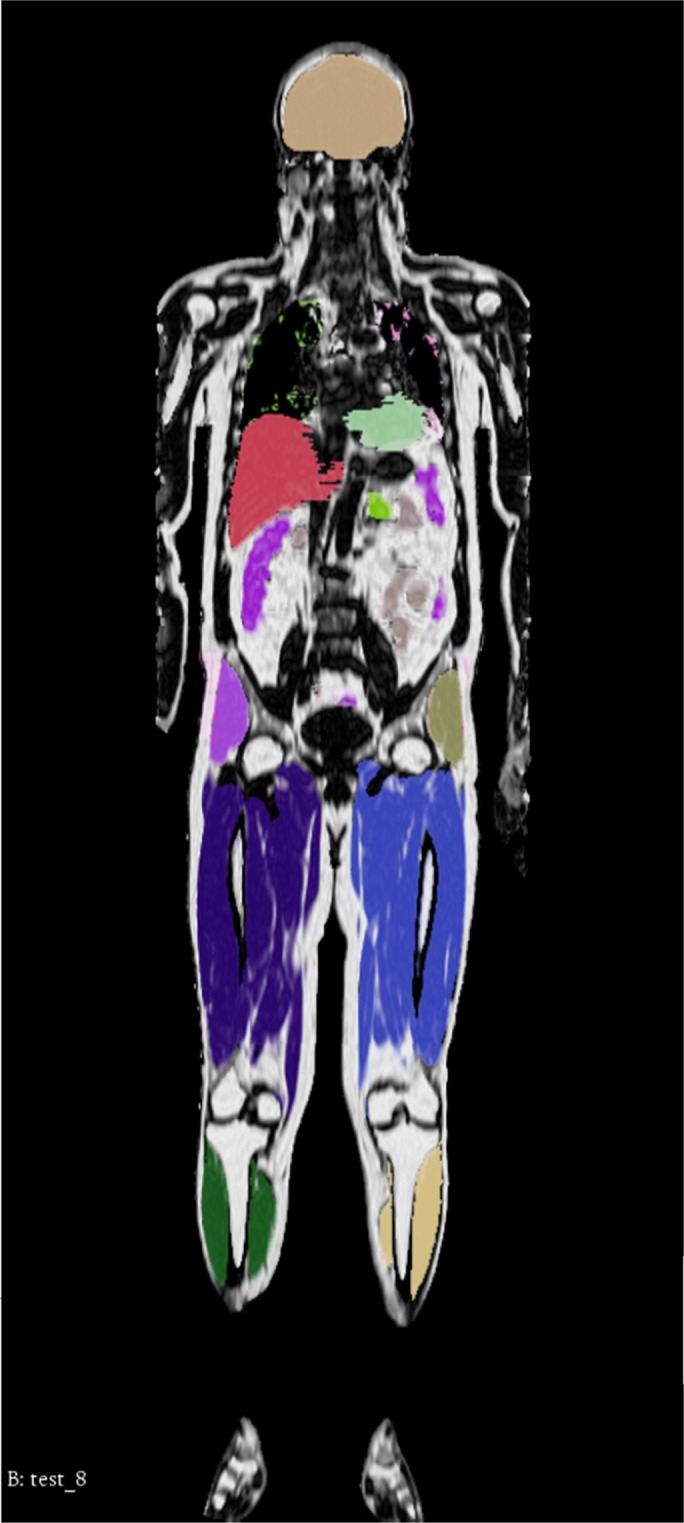
Illustration of reference tissue VOI segmentation. Each tissue evaluated has a different colour, also for left and right side. The colour are as follows: light beige (brain); light green (heart, left ventricle); red (liver); lime green (pancreas); purple (small intestine); beige (large intestine); light purple (right gluteal muscles); olive green (left gluteal muscles); blue (right thigh muscles); light blue (left thigh muscles); dark green (right calf muscles); yellow (left calf muscles). The other tissues are not shown in this slice.

This study comprised manual reference segmentation of a wide range of tissues (18 different VOIs) of 13 whole-body scans by a Radiologist with 5 years of experience, see Fig. 1. These include the reference person selected for the Imiomics pipeline as well as the 12 subjects selected for the validation. In case of uncertainty about a segmentation, especially in the case of intestines (small and large) delineation, the opinion of a second radiologist with 30 years of experience was requested.

The tissues evaluated in the analysis were: brain, including cerebellum (from the vertex to the last slice of the cerebellum hemispheres in coronal view); lungs (from apexes to bases in coronal view); heart (atria and ventricles in axial view); liver (from the dome to the last slice of the 6th segment in axial view); VAT (from the diaphragm to the sacral promontory in axial view); abdominal subcutaneous adipose tissue, SAT (analogous to VAT in axial view); pancreas (from the head to the tail in axial view); kidneys (from the upper pole to the lower pole in coronal view); bilateral gluteal muscles (maximus, medius and minimus in coronal view), thigh muscles (anterior, medial and posterior fascial compartments in sagittal view) and calf muscles (anterior, posterior and lateral compartments in sagittal view). The segmentations of the small and large intestines were performed by drawing the boundaries of the intestinal walls in axial view, after reaching a consensus between the two radiologists in the regions where the delineation of these organs was more equivocal, due to the overlap between intestinal loops and other anatomical structures or in the cases of breathing motion artifacts.

All the VOIs of the tissues were manually delineated on the MRAC images. All tissues but the adipose tissue VOIs (SAT and VAT) were identified using the water images. VAT and SAT were firstly delineated on fat-images and then filtered to only include voxels with a fat fraction at least of 50%, to avoid non-adipose tissue inclusion in the VOIs. For most segmentations the software 3D Slicer^21,22^, and the “Editor” tool was used. The manual VOI segmentation was mainly carried out in coronal views and corrections were made in cases of overlapping structures (e.g. lung/liver interface) in axial view. For SAT, thigh and calf muscle segmentation the program SmartPaint^23^ (Centre for Image Analysis, Uppsala University, Uppsala, Sweden), that allows interactive and time efficient volumetric segmentations of these tissues was used.

### Validation studies

The validation was performed by measurements of tissue volume, fat content and Ki in all tissues from both the reference and automated methods. For fat content and Ki both mean and median VOI voxel readouts were extracted and compared. The median readout might be more robust to small segmentation errors as it is less sensitive to outlier values from neighboring tissues with largely deviating intensities. In addition, the overlap between the VOIs of both methods was measured using the Dice score (Range 0–1 where 1 is the highest overlap). The VOI delineated in the subject space pre-registration was transformed to the reference space using the resulting deformation field of the registration and nearest neighbor interpolation. Dice scores was computed between the transformed VOI and the reference space VOI.

In addition to the average glucose uptake rate of a tissue we further quantified total tissue uptake rates (TTUR) to evaluate the accuracy of this automation. The reference liver- and pancreas fat measurements from the whole body MRAC data were compared to those from the explicit measurements from dedicated IDEAL scans using correlations and t-tests. Paired two-tailed t-tests were used to test for systematic differences between automated and reference measurements. Associations were analyzed in terms of R^2^ values, or coefficient of determination, that measures the proportion of the dependent variable that is predicted by the independent variable. P-values < 0.05 were considered significant. No correction for multiple testing was performed.

## Results

The main results are summarized in Table 2.

**Table 2:** Values obtained for Dice score, Fat fraction, Glucose Uptake Rate and Total Tissue Uptake Rate.

					Fat fraction (%)						Glucose Uptake Rate (Ki) x10–2						Total Tissue Upt Rate		
Tissue:	Dice	Tissue volume (L)			(Mean readouts)			(Median readouts)			(Mean readouts)			(Median readouts)			(TTUR)		
		Ref	Auto	R^2^	Ref	Auto	R^2^	Ref	Auto	R^2^	Ref	Auto	R^2^	Ref	Auto	R^2^	Ref	Auto	R^2^
**Brain**	0.92	**1.61**	1.53	0.926	—	—	—	—	—	—	1.89	**1.94**	0.991	1.93	**1.99**	0.991	**495.7**	481.0	0.994
**Lung R**	0.76	1.18	1.05	0.598	—	—	—	—	—	—	0.15	0.17	0.601	0.11	0.11	0.840	30.8	29.5	0.236
**Lung L**	0.71	0.87	0.80	0.500	—	—	—	—	—	—	0.26	0.30	0.414	0.15	0.16	0.770	34.9	37.6	0.091
**Heart**	0.77	0.47	0.48	0.434	11.7	**12.8**	0.969	8.4	9.0	0.953	3.13	3.08	0.828	2.12	1.99	0.341	234.4	226.1	0.905
**Liver**	0.82	1.84	1.83	0.688	11.5	**13.6**	0.902	8.9	**9.3**	0.993	0.60	0.58	0.981	0.56	0.55	0.984	178.9	171.3	0.972
**Kidney R**	0.76	**0.19**	0.14	0.083	13.5	**14.7**	0.930	9.9	**10.5**	0.927	—	—	—	—	—	—	—	—	—
**Kidney L**	0.75	**0.20**	0.17	0.386	13.5	**14.6**	0.703	9.7	**10.4**	0.844	—	—	—	—	—	—	—	—	—
**Pancreas**	0.36	0.09	**0.13**	0.693	28.2	**37.5**	0.859	23.9	**33.1**	0.815	0.34	0.35	0.842	0.31	0.31	0.912	4.7	**7.2**	0.708
**Small intestine**	0.34	**0.79**	0.62	0.182	19.4	**21.9**	0.914	17.1	**20.1**	0.887	0.72	0.72	0.023	**0.47**	0.41	0.538	92.4	80.6	0.007
**Large intestine**	0.32	0.44	0.43	0.067	19.3	**20.3**	0.449	17.7	18.1	0.408	0.78	**1.14**	0.255	0.61	0.63	0.906	60.2	91.7	0.005
**Subcut AT**	0.73	6.39	5.38	0.898	**90.4**	89.7	0.962	92.8	**95.3**	0.981	0.26	0.26	0.976	0.22	0.21	0.992	218.4	196.2	0.539
**Visceral AT**	0.87	4.37	4.54	0.945	**81.7**	80.0	0.984	**85.2**	83.3	0.988	0.44	0.43	0.995	0.35	0.35	0.996	272.4	279.4	0.965
**Gluteus muscles R**	0.84	1.59	1.51	0.561	24.4	25.1	0.724	18.5	18.3	0.956	1.35	1.35	0.972	1.21	1.22	0.968	346.1	326.1	0.982
**Gluteus muscles L**	0.85	1.57	1.67	0.657	24.7	**27.9**	0.848	18.6	**20.0**	0.962	**1.35**	1.29	0.992	1.24	1.18	0.984	342.7	340.7	0.994
**Thigh muscles R**	0.91	**3.56**	3.38	0.970	10.1	**10.8**	0.975	6.0	**6.7**	0.969	1.05	1.05	0.997	0.99	0.99	0.998	**617.8**	597.6	0.996
**Thigh muscles L**	0.93	3.60	3.59	0.961	9.8	**10.7**	0.976	5.5	**6.4**	0.960	1.05	1.04	0.998	0.98	0.97	0.998	619.3	624.5	0.997
**Calf muscles R**	0.91	**1.46**	1.34	0.976	9.5	**10.1**	0.977	6.8	**7.3**	0.996	1.24	1.24	0.991	1.21	1.22	0.987	**291.0**	271.7	0.998
**Calf muscles L**	0.91	**1.42**	1.28	0.971	8.9	**9.4**	0.939	6.1	**6.6**	0.976	1.18	1.17	0.994	1.15	1.13	0.986	**268.4**	247.9	0.991

Three different groups can be observed using the Dice scores as cut-off values, tissues that received high (<0.8), intermediate (0.7–0.8) and low scores (0.3–0.4). The brain, liver, VAT and lower-body muscles all received high Dice scores. The lungs, kidneys and SAT received intermediate scores and the pancreas, small and large intestine received low scores. The tissue volumes were significantly different between the reference and automated measurements in 8 out of 18 tissue depots. The mean absolute tissue volume difference of significant tissues was 16.7%, with the largest differences observed in the pancreas, kidneys and intestines. Out of the 8 significant volumetric differences 7 were due to underestimation. In lean tissues the fat fractions were slightly but significantly overestimated in all but one case, the average overestimation was +0.09 percentage points (pp). The median fat fraction readout had only a minor effect on the readout (3 non-significant tissues, average +0.09 pp).

The results from the dedicated liver fat scan (median readout) was 9.2 ± 6.2% and showed a correlation r = 0.956 and no sign diff (t-test p = 0.647) to the reference measures from whole-body MRI when a median readout was used. However, when a mean readout was used the results from whole-body MRI were higher (+2.3 pp, p = 0,005).

The results from the dedicated pancreas fat measurements acquired using dedicated imaging and manual segmentation resulted in FFs much lower than those from the reference measurements from whole-body MRI. (12.0 ± 8.7 < 23.9 ± 9.6, r = 0.877, p < 0.001).

The automated readout of Ki only deviated slightly and correlated strongly to the reference measures in most tissues. No clear systematic benefit from use of median readout mean readout was found. In the heart the median readout lowered the readout values significantly (p < 0.001 for both reference and automated measurements) and gave much lower R^2^ values between the reference and automated results.

## Discussion

We have shown that multiple features, including tissue volume, fat content and glucose uptake can be automatically quantified from whole-body PET-MRI datasets using Imiomics and a single image segmentation. The accuracy of most measurements is acceptable for automated extraction while certain tissues require an alternative approach or improved image registration quality.

The tissues showing the best performance were also generally the largest, including brain, liver, adipose tissue depots and muscle groups. These also show good contrast in water-fat MRI which benefits the image registration. They all show generally good performance in both Dice scores, estimated quantities and associations to the reference values.

As tissues and organs in the abdominal region are very challenging to register between subjects we did not expect very good results in this region. These tissues were rather included in this study to benchmark the estimation errors in these tissues and evaluate feasibility in large scale studies that might tolerate some loss in accuracy. Somewhat surprising was the finding that the features of the pancreas could be extracted with an R^2^ > 0.69 despite the significant differences in volume and fat fraction estimates and relatively low Dice score.

The automated volumetric estimations showed a wide range of correlations to the reference volume. The range in R^2^ were from 0.976 in muscles down to 0.067 in small intestine.

For fat fraction and glucose uptake both mean and median values were extracted and evaluated and no clear general systematic benefit from use of median compared to mean readout was found.

The brain volume is relatively accurately estimated with the automated approach. As the results are from a whole-body imaging protocol and much can be gained in accuracy and precision by dedicated brain MRI. The total brain volume (R^2^ = 0.93) and glucose uptake (R^2^ = 0.99) is however relatively accurately estimated. This good correlation is probably due to small variations in position between brains which simplifies the automated segmentations.

Lung tissue has very little signal in normal MRI and is here identified by low signal region. The MRAC images are also collected in free breathing and without cardiac triggering. Hence, images are affected by motion artifacts. The median readout of Ki seems preferential as it resulted in stronger correlations between reference and automated measurements.

The performed whole-body scanning in not optimal for heart imaging. Nevertheless, the automated analysis managed to estimate the heart characteristics relatively well. Median readout of Ki values gave much lower Ki values and weaker association between automated and reference measurements then readouts of the mean values. In the heart the left ventricle shows the largest Ki. As the total heart volume is much larger than the left ventricle the mean and median readouts show largely different quantities, inter subject variations and associations between reference and automated segmentation results. The relatively high fat fraction measured in the heart VOIs are not believed to be due to high myocardial fat contents but rather be caused by inclusion of pericardial fat in the measurements, due to partial volume effects and limitations in the segmentation quality.

The liver can vary largely in both size and shape. This makes in challenging to segment accurately using a single atlas approach. This is reflected in the volumetric measurements that show moderate correlation between reference an automated assessment. As the liver is relatively large intensity base measures of median fat fraction and Ki are however robust to segmentation errors. TTUR however, that includes the volumetric component, showed worse performance. For liver fat measurements the use of median value readouts seems preferable as this improved the correlation to the reference estimates. This is in line with our experience from previous studies and we typically apply median readouts from large liver volume segmentations in our liver fat quantification studies.

The water-fat scans (MRAC) used for estimations of fat fractions are so called 2-point Dixon scans as images at two echo times collected and used to model and estimate the MR water and fat signal. This allows high resolution scanning but at the prize of limited accuracy in estimation of fat and water content. However, a relatively high correlation between the MRAC and the state-of-the-art IDEAL-based quantifications of liver and pancreas fat was found.

The kidneys were segmented with a moderate accuracy (Dice around 0.75) and with an R^2^ for volume quantification of 0.08–0.39. This very poor correlation is believed to be due to a combination of limited segmentation quality and small variance in kidney volume (standard deviation of 0.042 L in all kidneys in the reference dataset). The automated segmentation is complicated by the fact that multiple lean tissues including intestines, liver, and spleen, might be present in the vicinity of the kidneys. The scans are also only mildly T1-weighted which limits the possibility to exploit tissue differences in T1 in the image registration. The kidney features of Ki and fat fraction are of limited interest as the Patlak model assumptions do not hold in kidney tissue and as the kidney parenchyma is not expected to contain any fat. The fat fraction estimated was surprisingly high (≥10%). This is most likely due to inclusion of surrounding adipose tissue due to partial volume effects.

The challenging task of segmenting the pancreas volume resulted in a Dice score of 0.36 in this evaluation. The R^2^ for pancreas volume estimation was however very similar to that of the liver volume. The Ki and the FFs also showed a relatively strong association to the reference values R^2^ > 0.7). The fat content was however significantly overestimated by approximately 10 pp with the automated analysis. The dedicated scanning and analysis also showed lower values. This is likely due to the fact that measurements from the dedicated scans carefully avoided the tissue borders to reduce partial volume effects to neighboring adipose tissue.

The intestines are challenging to accurately quantify from whole-body scans. Reasons include their varying content and their mobility especially of the small intestine. The associations in Ki and fat fraction using the median readouts (R^2^ > 0.4) show some promise for the possibility for application in large scale studies.

SAT & VAT show relatively good quantification accuracy for being achieved by a single image registration to a fixed reference subject. Dice scores are lower than previously presented for repeated manual analysis^24^ and also lower than previous example from dedicated imaging and analysis setup for abdominal fat quantifications^25^. The automated measurements from VAT appear more robust than those from SAT. This is likely because of challenges for image registration to handle the large interindividual variability in SAT between the sexes and the wide range of BMI included (24–39 kg/m^2^, SAT reference volume 1.5–12.8 L). The measured Ki:s in SAT in this study are lower than those in VAT which is in line with previous findings^10,26^.

The gluteus muscles could be quantified relatively accurately using this automated setup. They interestingly show high [^18^F]FDG uptake rate and more than twice as high fat fractions compared to the thigh and calf muscles. It is well known that glucose and [^18^F]FDG uptake rate is influenced by muscle fiber composition and muscle activity level^27^. Gluteal muscles were recently shown to be a highly reproducible location for measuring basal skeletal muscle [^18^F]FDG uptake rate with high intraclass correlation coefficient (ICC = 0.88) and low within-subject variation (2.2%)^28^. The increased uptake rate in these muscles can be explained by the fact that the gluteus maximus, one of the muscles that compose this group, is the biggest and strongest muscle of the whole body; moreover, their principal and most powerful function is to cause the body to regain the erect position after stopping and they are the chief muscles that work against gravity when a subject goes up stairs.

Thigh and calf muscles show high accuracy and strong prediction in all parameters. The performance of the automation is also better than for the gluteus muscles. This might be as both thigh and calf muscles have fewer neighboring tissues to compete with in the image registration.

The MRAC is the built-in scan for the system’s creation of attenuation correction maps. The MRAC is collected using whole-body coil with little intensity non-uniformities and always included as PET imaging is performed. These reasons made us set up the Imiomics pipeline using these scans. The images are typically collected on free breathing which gives some motion artifacts that limit image quality. The dynamic PET imaging is also affected by different sources of motion that limit the quantitative accuracy of the resulting images. These include respiratory, cardiac and as the total examination is about one hour it is also possible that small changes in positioning might occur. In this study these errors in the PET images affect both the reference and the automated approach.

Other study limitations include the limited number of subjects used in the reference creation. The reference creation by manual segmentation is indeed a challenging task that we try to avoid or limit. The careful manual segmentation work included in this study was approximately one month of full-time work (18 VOIs in 13 whole-body scans). There are possibilities to improve the image registration and measurement accuracy further in future studies. Possibilities include other strategies like for example deep learning for automated tissue segmentation. If accurate enough these could potentially be used directly for the measurements or to improve the image registration. It might also be possible to use the PET data acquired in the same session to guide and improve the image registration and/or segmentation. The multiple reference segmentations performed in this study can likely also be leveraged in future studies by for example multi-atlas segmentation approaches for improved accuracy. In this study we however chose to utilize a reference segmentation in a single subject reference space as we believe this is a relatively time efficient procedure to achieve various tissue segmentations that can be used in parallel with the voxel-wise analysis enabled by the Imiomics image analysis approach.

In conclusion, the automation of tissue identification and characterization works well in most metabolically relevant tissues suggesting the methodology to be useful in future quantification, especially in large-scale studies. Some tissues might for accuracy reasons benefit from other quantification methods or improvements in the current analysis approach.
